# Stress-Induced Hyperglycemia, but Not Diabetic Hyperglycemia, Is Associated with Higher Mortality in Patients with Isolated Moderate and Severe Traumatic Brain Injury: Analysis of a Propensity Score-Matched Population

**DOI:** 10.3390/ijerph14111340

**Published:** 2017-11-03

**Authors:** Cheng-Shyuan Rau, Shao-Chun Wu, Yi-Chun Chen, Peng-Chen Chien, Hsiao-Yun Hsieh, Pao-Jen Kuo, Ching-Hua Hsieh

**Affiliations:** 1 Department of Neurosurgery, Kaohsiung Chang Gung Memorial Hospital, Chang Gung University College of Medicine, Kaohsiung City 833, Taiwan; ersh2127@cloud.cgmh.org.tw; 2 Department of Anesthesiology, Kaohsiung Chang Gung Memorial Hospital, Chang Gung University College of Medicine, Kaohsiung City 833, Taiwan; shaochunwu@gmail.com; 3 Department of Plastic Surgery, Kaohsiung Chang Gung Memorial Hospital, Chang Gung University College of Medicine, Kaohsiung City 833, Taiwan; libe320@yahoo.com.tw (Y.-C.C.); VENU_CHIEN@hotmail.com (P.-C.C.); sylvia19870714@hotmail.com (H.-Y.H.); bow110470@gmail.com (P.-J.K.)

**Keywords:** traumatic brain injury (TBI), stress-induced hyperglycemia (SIH), diabetic hyperglycemia (DH), diabetes mellitus (DM), mortality

## Abstract

*Background*: Admission hyperglycemia is associated with higher morbidity and mortality in patients with traumatic brain injury (TBI). Stress-induced hyperglycemia (SIH), a form of hyperglycemia induced by the stress response, is associated with increased patient mortality following TBI. However, admission hyperglycemia occurs not only in SIH but also in patients with diabetic hyperglycemia (DH). Current information regarding whether trauma patients with SIH represent a distinct group with differential outcomes compared to those with DH remains limited. *Methods*: Serum glucose concentration ≥200 mg/dL upon arrival at the emergency department was defined as hyperglycemia. Presence of diabetes mellitus (DM) was determined by patient history and/or admission glycated hemoglobin (HbA1c) level ≥6.5%. In the present study, the patient cohort included those with moderate and severe TBI, as defined by an Abbreviated Injury Scale (AIS) score ≥3 points in the head, and excluded those who had additional AIS scores ≥3 points in any other region of the body. A total of 1798 adult patients with isolated moderate to severe TBI were allocated into four groups: SIH (*n* = 140), DH (*n* = 187), diabetic normoglycemia (DN, *n* = 186), and non-diabetic normoglycemia (NDN, *n* = 1285). Detailed patient information was retrieved from the Trauma Registry System at a level I trauma center between 1 January 2009, and 31 December 2015. Unpaired Student’s *t*- and Mann–Whitney *U*-tests were used to analyze normally and non-normally distributed continuous data, respectively. Categorical data were compared using the Pearson chi-square or two-sided Fisher’s exact tests. Matched patient populations were allocated in a 1:1 ratio according to propensity scores calculated by NCSS software. Logistic regression was used to evaluate the effect of SIH and DH on the adjusted mortality outcome. *Results*: In patients with isolated moderate to severe TBI, the presence of SIH and DH led to 9.1-fold and 2.3-fold higher odds of mortality, respectively, than patients with NDN. After adjusting for confounding factors, including sex and age, pre-existing co-morbidities, existence of different kinds of intracerebral hemorrhage, and injury severity, patients with SIH still had 6.6-fold higher odds of mortality than those with NDN; however, DH did not present significantly higher adjusted mortality odds. SIH and DH presented different effects on outcomes after TBI. The results also suggested that the pathophysiological effect associated with SIH was different from that of DH. *Conclusions*: This study demonstrated that patients with SIH and DH had significantly higher mortality than patients with NDN. However, the adjusted mortality was significantly higher only in the selected propensity score-matched patients with SIH and not in those with DH.

## 1. Introduction

Admission hyperglycemia is associated with higher morbidity and mortality in patients with traumatic brain injury (TBI) [1,2,3]. Patients with severe TBI have significantly higher serum glucose levels than those with mild TBI [1]. A stress response associated with TBI can induce a form of stress-induced hyperglycemia (SIH) [4,5,6], which also commonly occurs in patients with critical illnesses such as burn injuries [7], myocardial infarction [8], stroke [9,10], and trauma [11,12,13,14,15]. The hyperglycemia following TBI is suspected to contribute to tissue lactic acidosis in the brain and result in neuronal injury [16,17,18]. An association between SIH and increased mortality following TBI has been reported [5,19]. Many studies have also consistently shown SIH to be associated with higher morbidity and mortality rates in trauma patients [9,14,15,20].

However, admission hyperglycemia may occur not only as SIH but also as diabetic hyperglycemia (DH). Few studies have evaluated the differential effects of SIH and DH on the outcomes in the trauma population. Trauma patients with SIH have a >2-fold increase in mortality risk (relative risk (RR) 2.41, 95% confidence interval (CI) 1.81–3.23) than those without SIH, whereas patients with DH have a nonsignificant, near-50% increase in mortality risk (RR 1.47, 95% CI 0.92–2.36) [21]. Current information regarding whether patients with TBI having SIH represent a distinct group with different outcomes than those with DH remains limited. Bosarge et al. reported that patients with severe TBI and SIH had a 50% greater mortality (95% CI 1.13–1.95) than TBI patients with nondiabetic normoglycemia (NDN) after adjusting for age, sex, injury mechanism, Injury Severity Score (ISS), Revised Trauma Score (RTS), and lactic acid level [5]. On the other hand, DH patients did not have a significantly higher mortality [5]. However, this study did not exclude patients with polytrauma and did not compare the patients by adjusting for pre-existing co-morbidities and the existence of different kinds of intracerebral hemorrhage, which may impact the assessment of the mortality outcome. Therefore, the present study aimed to perform a further assessment of the effect of SIH and DH on the outcomes of the presence of hyperglycemia in patients with isolated moderate to severe TBI. We excluded patients with polytrauma and selected a propensity score-matched patient cohort to reduce the effect of differences in sex, age, pre-existing co-morbidities, and the existence of different kinds of intracerebral hemorrhage, such as epidural hemorrhage (EDH), subdural hemorrhage (SDH), subarachnoidal hemorrhage (SAH), and intracerebral hemorrhage (ICH), and injury severity in the patient population on the outcome assessment. The primary hypothesis of the present study was that patients with SIH had a similar or worse outcome than those with DH, based on the mortality rate as the primary measured outcome. 

## 2. Methods

### 2.1. Study Population

**Figure 1:**
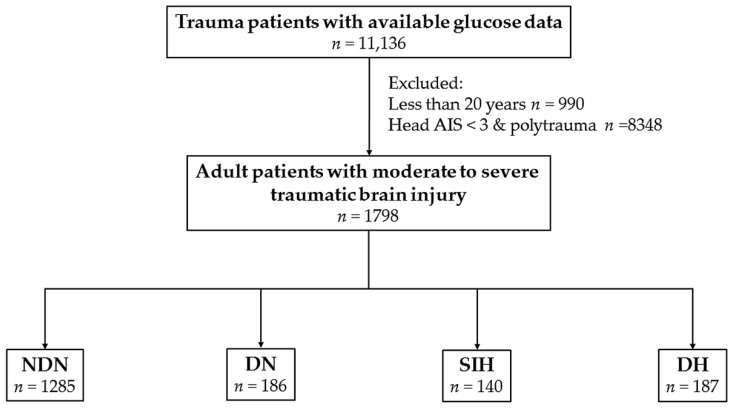
Flowchart of the allocation of patients with isolated moderate and severe traumatic brain injuries into four exclusive groups of non-diabetic normoglycemia (NDN), diabetic normoglycemia (DN), stress-induced hyperglycemia (SIH), and diabetic hyperglycemia (DH).

This study was approved by the Institutional Review Board (IRB) of the Kaohsiung Chang Gung Memorial Hospital, a Level I regional trauma center in southern Taiwan [22,23] before its implementation (reference number 201701210B0). According to IRB regulations, the requirement for informed consent was waived. The present retrospective study reviewed the data of all adult hospitalized trauma patients (≥20 years old) registered in the Trauma Registry System of the hospital from 1 January 2009 to 31 December 2015. In the present study, the patient cohort included those with moderate and severe TBI, defined by an Abbreviated Injury Scale (AIS) score ≥3 points in the head (AIS 3‒4 and 5 indicate moderate and severe TBI, respectively) [24]. To avoid the confounding effect of injuries to other body regions, polytrauma patients [25] with additional AIS scores ≥3 points in any other region of the body were excluded from the study; thus, the included patients were defined as having isolated moderate and severe TBI. In addition, only those patients with available data on serum glucose level in the emergency department as well as those patients with a history of diabetes mellitus (DM) or glycated hemoglobin (HbA1c) level data were included, whereas patients with incomplete data were excluded from the study. A serum glucose concentration ≥200 mg/dL upon arrival at the emergency department was defined as hyperglycemia. DM was diagnosed by patient history and/or admission HbA1c level ≥6.5%, according to the current recommendations for DM diagnosis from the American Diabetes Association [26]. SIH was diagnosed by serum glucose concentration ≥200 mg/dL in patients without DM and DH was diagnosed by serum glucose concentration ≥200 mg/dL in patients with DM. Diabetic normoglycemia (DN) and NDN was determined by serum glucose concentration <200 mg/dL in patients with and without DM, respectively. In the present study, the enrolled patients were allocated into four exclusive groups based on above definitions (Figure 1). The retrieved patient information for the study included the following: sex; age; existence of EDH, SDH, SAH, and ICH; co-morbidities such as hypertension (HTN), coronary artery disease (CAD), congestive heart failure (CHF), cerebral vascular accident (CVA), and end-stage renal disease (ESRD); serum glucose level at the emergency department; HbA1c level; Glasgow coma scale (GCS) scale; ISS, which was expressed as the median and interquartile range (IQR, Q1–Q3); hospital length of stay (LOS); rates of admission into the intensive care unit (ICU); and in-hospital mortality.

### 2.2. Statistical Analysis

We performed the statistical analyses using IBM SPSS Statistics for Windows, version 20.0 (IBM Corp., Armonk, NY, USA) and NCSS 10 software (NCSS Statistical Software, Kaysville, UT, USA). The primary outcome of the study was in-hospital mortality. Two-sided Fisher’s exact or Pearson chi-square tests were used to compare categorical data. Odds ratios (ORs) with 95% CIof the associated conditions of the patients were presented. The normally distributed continuous and non-normally distributed data were analyzed with unpaired Student’s *t*- and Mann–Whitney *U*-tests, respectively, and presented as mean ± standard deviation. To minimize the confounding effects of the baseline characteristics of the compared patient populations due to a non-randomized assignment, a 1:1 propensity score-matched study group was created using the Greedy method with a 0.2 caliper width using NCSS 10 software to assess the effect of SIH and DH on the outcomes. The propensity scores were calculated using a logistic regression model with the following covariates: sex, age, co-morbidities, types of intracranial hemorrhage, and ISS. After adjusting for these confounding factors, cox regression was used to evaluate the effects of SIH and DH on the primary and secondary outcomes against those of NDN. *p*-values < 0.05 were defined as statistically significant. 

## 3. Results

### 3.1. Characteristics and Outcomes of Patients with SIH

There were 1798 adult patients with isolated moderate to severe TBI in the present study (Figure 1). These patients were allocated into four groups: SIH (*n* = 140), DH (*n* = 187), DN (*n* = 186), and NDN (*n* = 1285). As shown in Table 1, no significant differences in sex and age were observed between the SIH and NDN groups. The SIH group had significantly higher rates of EDH, SDH, and ICH, but there was no difference between the rate of SAH in the SIH and NDN groups. No significant difference in the rates of pre-existing co-morbidities was observed between the SIH and NDN groups. On the other hand, more patients had a GCS ≤ 8 and fewer had a GCS score ≥ 13, the SIH group had a significantly lower GCS score than the NDN group (7.7 ± 4.7 vs. 12.4 ± 3.8, respectively; *p* < 0.001). The SIH group had a significantly higher ISS (median (IQR: Q1–Q3), 20 (16–25]) than the NDN group (16 (13–20]). In addition, more patients with SIH had an ISS ≥ 25, but fewer patients with SIH had an ISS < 16 than those with NDN. Regarding the patient outcomes, the SIH group had a higher proportion of patients admitted to the ICU (83.6% vs. 66.4%, respectively; *p* < 0.001) and 9.1-fold higher odds of mortality than the NDN group (95% CI 6.10–13.48; *p* < 0.001). There was no difference in the hospital LOS between the SIH and NDN groups.

### 3.2. Characteristics and Outcomes of Patients with DH

There was a significantly higher predominance of women and senior patients in the DH group than in the NDN group (Table 2). The DH group had a significantly higher rate of EDH than the NDN group, but no differences in the rates of SDH, SAH, and ICH were observed between the DH and NDN groups. Higher odds of HTN and CAD, but not other co-morbidities, were observed in the DH group than in the NDN group. There was no significant difference in GCS scores between the DH and NDN groups (12.1 ± 4.2 vs. 12.4 ± 3.8, respectively; *p* < 0.001), regardless of patient GCS score classifications of ≤8, between 9 and 12, and ≥13. There was no significant difference between the ISS (median (IQR: Q1–Q3), 16 (14–20) in the DH group and that in the NDN group (16 (13–20)). In addition, fewer DH patients had an ISS < 16 than in the NDN group. There was no difference in the hospital LOS and proportion of patients admitted to the ICU between the DH and NDN groups. However, patients in the DH group had 2.2-fold higher odds of mortality than those in the NDN group (95% CI 1.37–3.42; *p* = 0.001). 

### 3.3. Adjusted Outcomes of Patients with SIH and with DH

Propensity score-matched patients were selected to reduce the effect of differences in sex, age, pre-existing co-morbidities, types of intracranial hemorrhage, and injury severity of the patient population on the outcome assessment. As shown in Table 3, in selected 122 well-balanced pairs of SIH and NDN patients, there were no significant differences in sex, age, co-morbidity, types of intracranial hemorrhage, and ISS between the two patient cohorts. The logistic regression analysis of these pairs of patients showed that those with SIH had 6.6-fold higher odds of mortality (95% CI 2.58–16.91; *p* < 0.001) than those with NDN (test for coefficients mortality: b = 1.887; Wald = 15.462; *p* < 0.001, test for model coefficients: −2 Log Likelihood = 146.041, λ^2^ (1) = 20.632, *p* < 0.001). In addition, in the 163 well-balanced pairs of patients with DH and NDN (Table 4), there were also no significant differences in sex, age, co-morbidity, types of intracranial hemorrhage, and ISS between the two patient cohorts. The logistic regression analysis of these pairs of patients did not show a significant difference of odds of mortality (OR 1.4; 95% CI 0.68–2.71; *p* = 0.386) between DH and NDN (test for coefficients mortality: b = 0.305; Wald = 0.752; *p* = 0.386, test for model coefficients: −2 Log Likelihood = 225.205, λ^2^ (1) = 0.758, *p* = 0.384).

## 4. Discussion

In the present study of patients with isolated moderate to severe TBI, those with SIH and DH had 9.1-fold and 2.3-fold higher odds of mortality, respectively, than those with NDN. After adjusting for confounding factors, including sex and age, pre-existing co-morbidities, the existence of different kinds of intracerebral hemorrhage, and injury severity, patients with SIH still had 6.6-fold higher odds of mortality than those with NDN; however, the same was not observed in patients with DH, who did not present significantly higher mortality odds. This result was in accordance with the report by Bosarge et al. in which SIH in patients with severe TBI was associated with higher mortality than patients with NDN after adjusting for age, sex, injury mechanism, ISS, RTS, and lactic acid level; this increased mortality was not observed in those patients with DH [5]. Notably, in the present study, the adjusted 6.6-fold odds of mortality of SIH was much higher than that reported by Bosarge et al. who reported a 50% increased adjusted mortality in SIH patients with severe TBI [5]. We believe that the discrepancy may be partly attributed to the exclusion of patients with polytrauma from the present study. This discrepancy may also be partly attributed to the evaluation method with a propensity-score matched population by adjusting for pre-existing co-morbidities and different kinds of intracerebral hemorrhage. In the present study, the SIH group had a significantly higher ISS than the NDN and even the DH groups. The occurrences of a variety of intracerebral hemorrhages differed between the SIH and NDN groups and even between the SIH and DH groups. In addition, the patients with less severe injuries in the present study (moderate to severe TBI) than the population studied by Bosarge et al. (severe TBI) may also have contributed to the discrepancy, as the effect of hyperglycemia is less easy to measure in patients with high mortality. The above adjustments are expected to reduce bias in the assessment of mortality outcomes and infer a different extent of the odds of mortality. An adjustment to reduce bias in comparison is important during the assessment; for example, our previous study which explored the morbidities associated with SIH for all trauma patients had revealed that even the SIH had a 2.9-fold higher odds of pneumonia (95% CI 1.68–4.93; *p* < 0.001) and a 4.8-fold higher odds of acute renal failure (95% CI 2.15–10.52; *p* < 0.001) than the NDN, there were no differences in the rates of pneumonia and acute renal failure between SIH and NDN after adjustment by propensity-score matching [27].

Notably, hyperglycemia following TBI is associated with injury severity and poor outcomes [28,29]. Patients with severe TBI have significantly higher serum glucose levels than those with mild TBI [1]. The causes of SIH is secondary to a state of increased hepatic output of glucose, diminished insulin production, and insulin resistance in peripheral tissue in the presence of excessive adrenal cortical output (glucagon, growth hormone, catecholamine, and glucocorticoid) as well as high circulating levels of cytokines like tumor necrosis factor-α, interleukin-1, and interleukin-6 [20,30]. Up to 10 times of these greater adrenal cortical outputs causing SIH was found [31]. In contrast, DH is a chronic process associated with microvascular changes due to prolonged hyperglycemia [20]. In the present study, SIH and DH differed in their effect on the outcomes of patients after TBI. Although the mechanism for the detrimental effects of these two hyperglycemia states is unknown, the results indicate that the pathophysiological effect associated with SIH might be different from that of DH. In this study, the SIH could be provided as a marker for worse mortality. However, whether SIH is directly harmful to the subject remained debatable. Historically, SIH appears to be a marker of disease severity and the hyperglycemia and insulin resistance induced in the setting of acute illness had been thought to be a protective mechanism that increases the host’s chances of survival [31]. In experimental animal models, the results are inconsistent to determine whether SIH is harmful, neutral, or beneficial for outcomes following TBI [32,33]. Furthermore, currently no definitive statements can be made as to whether aggressive treatment of hyperglycemia actually benefits outcomes of trauma patients [34,35,36].

The present study had several limitations. The first limitation is represented by the inherent selection bias associated with the retrospective study design. The cause of mortality of these patients with TBI was unknown in this retrospective study, and thus may result in a selection bias in the comparison of outcomes between groups. Second, patients declared dead at the scene of the accident or upon hospital arrival were not included in the database, which may have led to a selection bias, considering that the mortality rate was the primary outcome. Third, as patients with DH may have some degree of stress response invoking their hyperglycemia, SIH and DH are not mutually exclusive. Currently, the SIH in diabetes has not been explored and lacks a well-established definition. In addition, because we did not measure the levels of stress response hormones or catecholamine, it was not possible for us to specifically identify whether stress might be more responsible for the hyperglycemia in the patients with DH. Furthermore, the use of glycemic control is not performed under strict guidelines at our hospital and may vary among patients with TBI. In addition, some diabetes drugs targeting the glucagon-like peptide-1 receptor, such as metformin and thiazolidinediones, may promote neuronal survival by affecting brain metabolism, neuroinflammation, and regeneration [37]. However, their effect on mortality could not be corrected in this retrospective study, and thus may result in a bias in the outcome assessment. Although the studies on the use and effect of glycemic control have been inconclusive, this may have resulted in a bias in the outcome assessment. This also indicates that defining the appropriate treatment for SIH remains an area of ongoing investigation. 

## 5. Conclusions

The results of this study demonstrated worse outcomes for patients with stress-induced hyperglycemia (SIH), but not diabetic hyperglycemia (DH), among patients with isolated moderate to severe traumatic brain injury (TBI) with respect to mortality, after controlling for age, sex, pre-existing co-morbidities, the existence of different kinds of intracerebral hemorrhage, and Injury Severity Score (ISS).

## Figures and Tables

**Table 1:** Comparison of the characteristics and outcomes of the SIH and NDN groups of patients.

Variables	SIH *n* = 140	NDN *n* = 1285	Odds Ratio (95% CI)	*p*
Sex				0.984
Female, *n* (%)	50 (35.7)	460 (35.8)	1.0 (0.69–1.43)	
Male, *n* (%)	90 (64.3)	825 (64.2)	1.0 (0.70–1.44)	
Age (years)	52.4 ± 18.7	54.3 ± 19.8	-	0.297
Hemorrhage type				
EDH, *n* (%)	45 (32.1)	273 (21.2)	1.8 (1.20–2.57)	0.003
SDH, *n* (%)	90 (64.3)	688 (53.5)	1.6 (1.09–2.25)	0.015
SAH, *n* (%)	76 (54.3)	634 (49.3)	1.2 (0.86–1.73)	0.266
ICH, *n* (%)	30 (21.4)	137 (10.7)	2.3 (1.47–3.55)	<0.001
Co-morbidity				
HTN, *n* (%)	36 (25.7)	326 (25.4)	1.0 (0.68–1.52)	0.929
CAD, *n* (%)	4 (2.9)	42 (3.3)	0.9 (0.31–2.47)	1.000
CHF, *n* (%)	2 (1.4)	8 (0.6)	2.3 (0.49–11.00)	0.257
CVA, *n* (%)	2 (1.4)	55 (4.3)	0.3 (0.08–1.34)	0.102
ESRD, *n* (%)	0 (0.0)	2 (0.2)	-	1.000
GCS	7.7 ± 4.7	12.4 ± 3.8	-	<0.001
≤8	89 (63.6)	255 (19.8)	7.0 (4.87–10.21)	<0.001
9–12	18 (12.9)	157 (12.2)	1.1 (0.63–1.79)	0.827
≥13	33 (23.6)	873 (67.9)	0.1 (0.10–0.22)	<0.001
ISS, median (IQR)	20 (16–25)	16 (13–20)	-	<0.001
<16	23 (16.4)	466 (36.3)	0.3 (0.22–0.55)	<0.001
16–24	65 (46.4)	678 (52.8)	0.8 (0.55–1.10)	0.154
≥25	52 (37.1)	141 (11.0)	4.8 (3.26–7.05)	<0.001
Hospital LOS (days)	14.4 ± 21.1	12.2 ± 12.5	-	0.236
ICU admission, *n* (%)	117 (83.6)	853 (66.4)	2.6 (1.62–4.09)	<0.001
Mortality, *n* (%)	58 (41.4)	93 (7.2)	9.1 (6.10–13.48)	<0.001

CAD = coronary artery disease; CHF = congestive heart failure; CI = confidence interval; CVA = cerebral vascular accident; EDH = epidural hemorrhage; ESRD = end-stage renal disease; GCS = Glasgow coma scale; HTN = hypertension; ICH = intracerebral hemorrhage; ICU = intensive care unit; IQR = interquartile range; ISS = injury severity score; LOS = length of stay; NDN = nondiabetic normoglycemia; SAH = subarachnoid hemorrhage; SDH = subdural hemorrhage; SIH = stress-induced hyperglycemia.

**Table 2:** Comparison of the characteristics and outcomes of the DH and NDN groups of patients.

Variables	DH *n* = 187	NDN *n* = 1285	Odds Ratio (95% CI)	*p*
Sex				<0.001
Female, *n* (%)	92 (49.2)	460 (35.8)	1.7 (1.28–2.37)	
Male, *n* (%)	95 (50.8)	825 (64.2)	0.6 (0.42–0.78)	
Age (years)	65.6 ± 12.0	54.3 ± 19.8	-	<0.001
Hemorrhage type				
EDH, *n* (%)	25 (13.4)	273 (21.2)	0.6 (0.37–0.89)	0.012
SDH, *n* (%)	104 (55.6)	688 (53.5)	1.1 (0.80–1.48)	0.595
SAH, *n* (%)	78 (41.7)	634 (49.3)	0.7 (0.54–1.00)	0.051
ICH, *n* (%)	22 (11.8)	137 (10.7)	1.1 (0.69–1.80)	0.650
Co-morbidity				
HTN, *n* (%)	116 (62.0)	326 (25.4)	4.8 (3.49–6.63)	<0.001
CAD, *n* (%)	20 (10.7)	42 (3.3)	3.5 (2.03–6.18)	<0.001
CHF, *n* (%)	4 (2.1)	8 (0.6)	3.5 (1.04–11.70)	0.055
CVA, *n* (%)	14 (7.5)	55 (4.3)	1.8 (0.99–3.32)	0.053
ESRD, *n* (%)	0 (0.0)	2 (0.2)	-	1.000
GCS	12.1 ± 4.2	12.4 ± 3.8	-	0.320
≤8	44 (23.5)	255 (19.8)	1.2 (0.86–1.79)	0.242
9–12	19 (10.2)	157 (12.2)	0.8 (0.49–1.34)	0.418
≥13	124 (66.3)	873 (67.9)	0.9 (0.67–1.29)	0.656
ISS, median (IQR)	16 (14–20)	16 (13–20)	-	0.183
<16	50 (26.7)	466 (36.3)	0.6 (0.46–0.90)	0.011
16–24	108 (57.8)	678 (52.8)	1.2 (0.90–1.67)	0.201
≥25	29 (15.5)	141 (11.0)	1.5 (0.97–2.30)	0.070
Hospital LOS (days)	13.3 ± 12.0	12.2 ± 12.5	-	0.296
ICU admission, *n* (%)	134 (71.7)	853 (66.4)	1.3 (0.91–1.80)	0.151
Mortality, *n* (%)	27 (14.4)	93 (7.2)	2.2 (1.37–3.42)	0.001

CAD = coronary artery disease; CHF = congestive heart failure; CI = confidence interval; CVA = cerebral vascular accident; DH = diabetic hyperglycemia; EDH = epidural hemorrhage; ESRD = end-stage renal disease; GCS = Glasgow coma scale; HTN = hypertension; ICH = intracerebral hemorrhage; ICU = intensive care unit; IQR = interquartile range; ISS = injury severity score; LOS = length of stay; NDN = nondiabetic normoglycemia; SAH = subarachnoid hemorrhage; SDH = subdural hemorrhage.

**Table 3:** Comparison of the mortality outcomes in the selected propensity-score matched cohort of SIH and NDN groups of patients.

Variables	Propensity Score-Matched Cohort				
	SIH *n* = 122	NDN *n* = 122	Odds Ratio (95% CI)	*p*	Standardized Difference
Sex				1.000	
Female, *n* (%)	40 (32.8)	40 (32.8)	1.0 (0.59–1.71)		0.00%
Male, *n* (%)	82 (67.2)	82 (67.2)	1.0 (0.59–1.71)		0.00%
Age (years)	50.9 ± 18.7	51.0 ± 17.9	-	0.992	−0.13%
Co-morbidity					
HTN, *n* (%)	25 (20.5)	25 (20.5)	1.0 (0.54–1.86)	1.000	0.00%
CAD, *n* (%)	1 (0.8)	1 (0.8)	1.0 (0.06–16.17)	1.000	0.00%
CHF, *n* (%)	0 (0.0)	0 (0.0)	-	-	-
CVA, *n* (%)	1 (0.8)	1 (0.8)	1.0 (0.06–16.17)	1.000	0.00%
ESRD, *n* (%)	0 (0.0)	0 (0.0)	-	-	-
EDH, *n* (%)	42 (34.4)	42 (34.4)	1.0 (0.59–1.70)	1.000	0.00%
SDH, *n* (%)	83 (68.0)	83 (68.0)	1.0 (0.58–1.71)	1.000	0.00%
SAH, *n* (%)	63 (51.6)	63 (51.6)	1.0 (0.61–1.65)	1.000	0.00%
ICH, *n* (%)	21 (17.2)	21 (17.2)	1.0 (0.51–1.94)	1.000	0.00%
ISS, median (IQR)	17 (16–25)	17 (16–25)	-	0.898	0.00% ^†^
Mortality, *n* (%)	49 (40.2)	21 (17.2)	6.6 (2.58–16.91)	<0.001	

CAD = coronary artery disease; CHF = congestive heart failure; CI = confidence interval; CVA = cerebral vascular accident; EDH = epidural hemorrhage; ESRD = end-stage renal disease; HTN = hypertension; ICH = intracerebral hemorrhage; IQR = interquartile range; ISS = injury severity score; NDN = nondiabetic normoglycemia; SAH = subarachnoid hemorrhage; SDH = subdural hemorrhage; SIH = stress-induced hyperglycemia. ^†^ indicates difference of interquartile range.

**Table 4:** Comparison of mortality outcomes in the selected propensity-score matched cohort of DH and NDN groups of patients.

Variables	Propensity Score-Matched Cohort				
	DH *n* = 163	NDN *n* = 163	Odds Ratio (95% CI)	*p*	Standardized Difference
Sex				1.000	
Female, *n* (%)	74 (45.4)	74 (45.4)	1.0 (0.65–1.55)		0.00%
Male, *n* (%)	89 (54.6)	89 (54.6)	1.0 (0.65–1.55)		0.00%
Age (years)	65.0 ± 12.3	65.3 ± 13.1	-	0.852	−2.07%
Co-morbidity					
HTN, *n* (%)	97 (59.5)	97 (59.5)	1.0 (0.64–1.56)	1.000	0.00%
CAD, *n* (%)	6 (3.7)	6 (3.7)	1.0 (0.32–3.17)	1.000	0.00%
CHF, *n* (%)	1 (0.6)	1 (0.6)	1.0 (0.06–16.13)	1.000	0.00%
CVA, *n* (%)	11 (6.7)	11 (6.7)	1.0 (0.42–2.38)	1.000	0.00%
ESRD, *n* (%)	0 (0.0)	0 (0.0)	-	-	-
EDH, *n* (%)	21 (12.9)	21 (12.9)	1.0 (0.52–1.91)	1.000	0.00%
SDH, *n* (%)	87 (53.4)	87 (53.4)	1.0 (0.65–1.55)	1.000	0.00%
SAH, *n* (%)	69 (42.3)	69 (42.3)	1.0 (0.64–1.55)	1.000	0.00%
ICH, *n* (%)	15 (9.2)	15 (9.2)	1.0 (0.47–2.12)	1.000	0.00%
ISS, median (IQR)	16 (13–20)	16 (13–20)	-	0.916	0.00% ^†^
Mortality, *n* (%)	23 (14.1)	18 (11.0)	1.4 (0.68–2.71)	0.386	

CAD = coronary artery disease; CHF = congestive heart failure; CI = confidence interval; CVA = cerebral vascular accident; DH = diabetic hyperglycemia; EDH = epidural hemorrhage; ESRD = end-stage renal disease; HTN = hypertension; ICH = intracerebral hemorrhage; IQR = interquartile range; ISS = injury severity score; NDN= nondiabetic normoglycemia; SAH = subarachnoid hemorrhage; SDH = subdural hemorrhage. ^†^ indicates difference of interquartile range.
